# Prolonged Ventilator Dependency in a Pediatric ICU Patient With Pfeiffer Syndrome Following Le Fort I Osteotomy and Distraction Osteogenesis: A Case Report

**DOI:** 10.7759/cureus.71617

**Published:** 2024-10-16

**Authors:** Christy Lee, Zachrieh Alhaj, Zaid Almubaid, Nikhita Kichili, Sharif Mohamed

**Affiliations:** 1 Anesthesiology, University of Texas Medical Branch, Galveston, USA

**Keywords:** le fort 1 osteotomy, pediatric icu, pediatric sedation, pfeiffer syndrome, prolonged mechanical ventilation

## Abstract

Pfeiffer syndrome is a rare autosomal dominant disorder characterized by craniosynostosis and midface hypoplasia, often leading to significant airway challenges and respiratory complications during anesthesia management. This case report describes a four-year-old female with Pfeiffer syndrome who underwent Le Fort I osteotomy with distraction osteogenesis and concurrent ventriculoperitoneal shunt revision. Postoperatively, the patient experienced an extended pediatric ICU (PICU) stay with complex airway management, requiring prolonged mechanical ventilation and sedation. This case highlights the complexities in managing patients with Pfeiffer syndrome undergoing craniofacial surgery and emphasizes the importance of early sedation weaning, sedation windows, use of shorter active sedatives, timely tracheostomy, and multidisciplinary education to improve respiratory outcomes.

## Introduction

Pfeiffer syndrome is a rare autosomal dominant disorder characterized by craniosynostosis, midface hypoplasia, and broad, short thumbs. Occasionally, it can also be associated with hydrocephalus, ocular proptosis, ankylosed elbows, and delayed growth [1,2]. It is caused by mutations in the FGFR1 or FGFR2 genes, which lead to abnormal bone growth [1]. The prevalence of this condition is about 1 in 100,000 individuals [1]. In anesthesia management, patients with Pfeiffer syndrome often present with a range of complications, including respiratory distress due to midface hypoplasia and airway obstruction. Surgical interventions are commonly required to address these anatomical abnormalities, with midface elongation procedures being an essential treatment approach.

In this case report, we discuss the case of a pediatric patient who underwent Le Fort I osteotomy and distraction osteogenesis. Postoperatively, our patient had a prolonged stay in the ICU and remained dependent on mechanical ventilation and sedation for over a month.

## Case presentation

A four-year-old female with a known diagnosis of Pfeiffer syndrome, with midface hypoplasia causing obstructive sleep apnea (OSA), presented for a Le Fort I osteotomy with distraction osteogenesis and concurrent ventriculoperitoneal shunt revision. Other manifestations of her syndrome included craniosynostosis, small atrial septal defect (ASD), choanal stenosis, exposure keratopathy, cloverleaf skull, and hypoplasia of the mandible and maxilla.

Before the surgery, the anesthesia team encountered a difficult intubation. Two attempts were required, necessitating video laryngoscopy (C-MAC). Intraoperatively, the patient tolerated the procedure well. Postoperatively, the patient was transferred to the pediatric ICU (PICU), intubated, and sedated on propofol and fentanyl infusions.

Throughout her PICU stay, extubation was delayed due to several factors, including severe tongue edema, respiratory compromise secondary to COVID-19 infection, *Pseudomonas *tracheitis requiring antibiotic therapy, and fluid overload with resultant abdominal distension. The patient remained intubated and sedated for 18 days postoperatively. On postoperative day (POD) 2, the patient was started on a midazolam drip for agitation control. Due to jaw clenching, a vecuronium infusion was initiated on POD 9. On POD 18, the patient was taken back to the operating room for a tracheostomy due to ongoing respiratory insufficiency and failure to wean from mechanical ventilation.

Following tracheostomy placement, her course remained complex, with difficulty weaning from the ventilator and frequent tracheostomy displacement due to sensitive positioning. The patient remained on dexmedetomidine and propofol for sedation for an additional 15 days following the tracheostomy, bringing her total sedation period to 33 days. During this time, she also required vasopressor support for hemodynamic instability.

## Discussion

This case illustrates a prolonged PICU stay for a pediatric patient with Pfeiffer syndrome following Le Fort I osteotomy and distraction osteogenesis. Le Fort I osteotomy is a surgical procedure that corrects midface hypoplasia by surgically detaching the skull base and repositioning it forward. Distraction osteogenesis is often used in conjunction with the previous procedure to lengthen the bone by applying tension to the osteotomy site, helping to promote bone growth [3]. Patients with Pfeiffer syndrome present many challenges in operative management due to underlying abnormalities. As such, Pfeiffer patients are more likely to experience airway difficulties and respiratory complications during or after surgery [4].

In this case, the patient’s airway compromise, postoperative complications, and infections prolonged her mechanical ventilation. Our patient had midface hypoplasia due to Pfeiffer syndrome, which caused her to develop OSA. Postoperatively, she experienced oropharyngeal and tongue edema complications, which worsened her OSA. Airway edema is a common complication in craniofacial surgery and can prolong the need for ventilatory support, which occurred in this case [5]. In addition to her airway-related complications, the patient’s postoperative course was further complicated by concurrent infections, including COVID-19 and *Pseudomonas* tracheitis, which likely prolonged her recovery. Infections such as these are known to extend the duration of mechanical ventilation and the requirement for increased sedation, as seen in this case [6].

To optimize perioperative management in similar cases, several considerations could be explored to prevent or mitigate complications like those experienced by this patient. One potential strategy is earlier sedation weaning. Our patient received prolonged infusions of sedatives, including propofol, dexmedetomidine, midazolam, morphine, and methadone, which likely contributed to delayed extubation. Early weaning of sedation could reduce the duration of mechanical ventilation and lower the risk of sedation-related complications, such as hypotension, neurologic deficits, and withdrawal symptoms [7]. Additionally, implementing "sedation windows," where sedation is intermittently minimized to assess the patient’s respiratory function, could help identify the optimal timing for extubation [8]. Early tracheostomy may also be beneficial in cases where prolonged intubation is anticipated. In this case, a tracheostomy was performed on POD 18, after the patient had demonstrated significant difficulty with extubation. An earlier tracheostomy could have potentially shortened the duration of mechanical ventilation and improved respiratory outcomes. Furthermore, shorter-acting sedatives with more predictable pharmacokinetics may facilitate faster sedation adjustments, reducing the risks associated with prolonged sedation [9].

**Figure 1:**
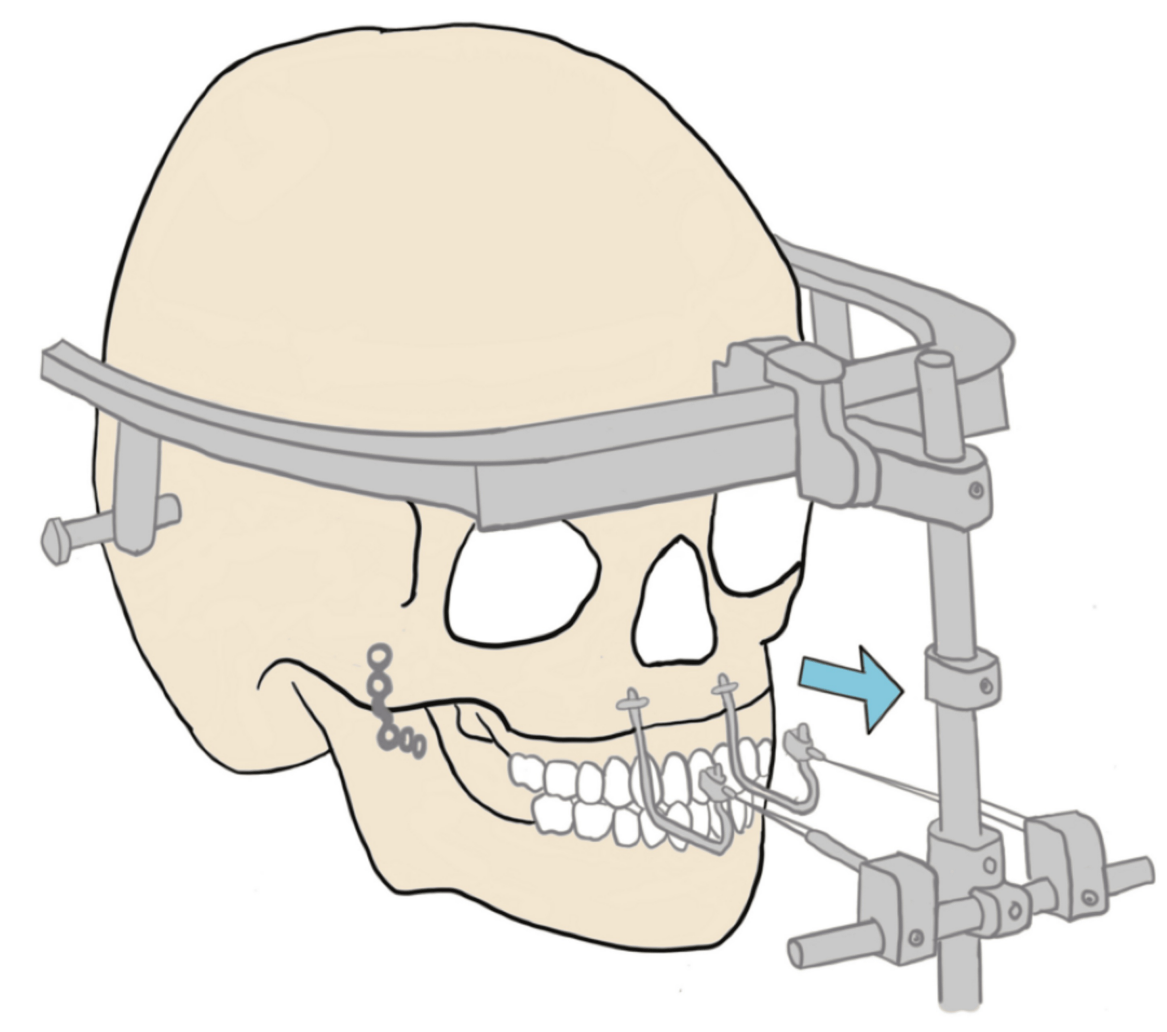
Diagram of the external fixation hardware placed after the patient’s Le Fort osteotomy, highlighting the challenges it poses for accessing the patient’s airway. Image credit: Dr. Christy Lee The arrow indicates the direction in which the external hardware was pulling as part of the Le Fort osteotomy procedure.

This case also highlights the technical challenges posed by the external fixator placed after the Le Fort I osteotomy. While essential for distraction osteogenesis, the fixator complicates respiratory care by limiting access to the patient’s airway (Figure 1). Given the patient's history of difficult intubation, choanal stenosis, and macroglossia, maintaining secure airway access was critical. The need for a specialized screwdriver and wire cutter to remove the fixator in an emergency further increased the complexity of airway management. To mitigate this risk in future cases, thorough training of PICU staff in removing external fixators and clearly documented reintubation protocols are essential.

## Conclusions

This case highlights the multifactorial challenges faced when providing care to patients with Pfeiffer syndrome undergoing major craniofacial surgery. In our case, several variables and complications increased the amount of time our patient remained dependent on the ventilator. Further research is needed to improve the operative care of patients, especially those with underlying conditions such as Pfeiffer syndrome, and to enhance ventilation strategies and outcomes. A multidisciplinary approach is also important to improve outcomes and reduce the risk of prolonged ventilatory support and complications.
